# Immunogenicity of Protein Therapeutics: A Lymph Node Perspective

**DOI:** 10.3389/fimmu.2020.00791

**Published:** 2020-05-14

**Authors:** Kristy Fu, Kylie March, Aikaterini Alexaki, Giulia Fabozzi, Eirini Moysi, Constantinos Petrovas

**Affiliations:** ^1^Tissue Analysis Core, Immunology Laboratory, Vaccine Research Center, NIAID, National Institutes of Health (NIH), Bethesda, MD, United States; ^2^Center for Biologics Evaluation and Research, Food and Drug Administration, Silver Spring, MD, United States

**Keywords:** therapeutics, follicle, germinal center, B cells, ADA, Tfh cell

## Abstract

The continuous development of molecular biology and protein engineering technologies enables the expansion of the breadth and complexity of protein therapeutics for *in vivo* administration. However, the immunogenicity and associated *in vivo* development of antibodies against therapeutics are a major restriction factor for their usage. The B cell follicular and particularly germinal center areas in secondary lymphoid organs are the anatomical sites where the development of antibody responses against pathogens and immunogens takes place. A growing body of data has revealed the importance of the orchestrated function of highly differentiated adaptive immunity cells, including follicular helper CD4 T cells and germinal center B cells, for the optimal generation of these antibody responses. Understanding the cellular and molecular mechanisms mediating the antibody responses against therapeutics could lead to novel strategies to reduce their immunogenicity and increase their efficacy.

## Introduction

Protein therapeutics is a new class of drugs that, unlike small molecule drugs, are not chemically synthesized; instead, they are produced within living cells or organisms. Remarkable developments in molecular biology and protein engineering methodologies in the last few decades have enabled the generation of several new biotherapeutics for a wide range of diseases. Despite the potential of protein therapeutics, a drawback, often associated with them, is the generation of antidrug antibodies (ADAs), which diminishes the bioactivity and effectiveness of the therapeutic (1). The anatomical sites where the development of ADA occurs are the secondary lymphoid organs, including lymph nodes and spleen, which are central for humoral responses to immunogens and pathogens (2–4). The organogenesis (5, 6), architecture (7, 8), and cellular composition (9, 10) of lymph nodes as well as the immune reactions to pathogens mediating the development of humoral responses (11, 12) are well-studied and understood. Here, we review these principles with a focus on ADA development and potential strategies aiming to minimize the immunogenicity of biotherapeutics.

## Historical Background

The history of protein therapeutics starts probably with diphtheria antitoxin derived from horse serum. The extraction of insulin from bovine pancreas, a few decades later, was a milestone in the treatment of diabetes. The development and approval of recombinant insulin, a human insulin expressed in *Escherichia coli*, in 1982 (13) resulted in the increased accessibility of insulin. Around the same time, murine monoclonal antibodies were being considered as therapeutic agents, with Orthoclone OKT3 (muromonab-CD3) being the first licensed monoclonal antibody, in 1986 (14). Many of the first-generation monoclonal antibodies were significantly immunogenic, because of their murine origin. Orthoclone OKT3 was eventually discontinued from the market in 2010 owing to the development of ADA (14–17). New generations of monoclonal antibodies are primarily being developed as humanized or human antibodies and are far less immunogenic. The use of a fully humanized IgG1 mAb against TNFα (adalimumab) triggered the development of ADA in patients with plaque psoriasis (18) and rheumatoid arthritis (19–22), indicating that ADA development to therapeutics can vary depending on factors like preexisting activation of the immune system and chronic inflammation.

Monoclonal antibodies constitute a large fraction of biotherapeutics and target a specific protein, usually to inhibit or modulate its function; in some cases, they may have a diagnostic role, or they may deliver a drug. The rest of protein therapeutics are primarily replacement therapies for proteins that are deficient owing to genetic or other reasons. Such protein therapeutics include coagulation factors, hormones, growth factors, and enzymes. Recently, fusion proteins are becoming an important class of biotherapeutics (Table 1). There are several examples of albumin fusion, Fc fusion, and antibody drug conjugates available in the market (33). The breadth and the complexity of protein therapeutics increase the potential for immune response generation. Protein therapeutic immunogenicity poses a great challenge in the field; and a better understanding of the risks, development, and mechanisms of ADA is needed to allow for strategies to reduce immunogenicity.

**Table 1:** Reported ADA development for licensed therapeutics.

**Drug name**	**Type**	**Clinical use**	**ADA prevalence**	**Citation**
**Monoclonal antibodies**				
Muromonab-CD3	Murine against CD3	Immunosuppression for the prevention of allograft rejection in transplants	43–91%	(14–17)
Infliximab	Chimeric human/mouse IgG1 against TNFα	Rheumatoid arthritis, inflammatory bowel disease, plaque psoriasis	5.4–43.6%	(19, 21, 23)
Cetuximab	Chimeric human/mouse IgG1 against EGFR	Colorectal cancer, squamous cell carcinoma (head/neck)	0.6–20.8%	(24)
Adalimumab	Human IgG1 against TNFα	Plaque psoriasis, rheumatoid arthritis, Crohn's disease, spondyloarthritis, psoriatic arthritis	17–49%	(18, 20–22, 25)
**Other drugs**				
L-Asparaginase	Enzyme	Acute lymphoblastic leukemia in adult and children	2% (PEG-Asp–neutralizing ADA;) 26% (*Escherichia coli*-Asp)	(26, 27)
FVIII	Anti-hemorrhagic protein	Hemophilia A/B	3.6–33% (25–30% in those with severe hemophilia)	(28, 29)
IFN-β	Mammalian cytokine	Multiple sclerosis	2–53%	(30–32)

*ADA, antidrug antibody; EGFR, epidermal growth factor receptor*.

### Generation and Mechanisms of Action of Antidrug Antibodies

Several factors contributing to the immunogenicity of biotherapeutics have been identified and in many cases eliminated to generate better drugs. For example, animal proteins have been for the most part phased out of the market, as these were very often associated with strong immune responses. Initially, many recombinant proteins were generated through bacterial systems (especially *E. coli*), which are effective and simple to use but lack the higher mechanisms for glycosylation and therefore are often immunogenic in humans. Mammalian expression systems are now commonly used for biotherapeutics to allow for glycosylation. However, even within mammalian species, glycosylation may differ contributing to the development of ADA. For example, cetuximab, a monoclonal antibody against epidermal growth factor receptor (EGFR) inhibitor (24, 34, 35) generated in a mouse myeloma cell line SP2/0 was associated with development of ADA owing to its glycosylation profile (24, 34–36). Introduction of alternative mammalian cell lines [Chinese hamster ovary (CHO)] has greatly contributed to overcoming immunogenicity due to glycosylation patterns (34, 35). l-Asparaginase, a highly immunogenic enzyme, is effective in its own native, *E. coli* derived form (37, 38). However, allergic reactions due to multiple doses caused silent hypersensitivity that in turns generates ADA. Use of a pegylated form (26) or increasing the enzyme binding to erythrocytes (39) was able to reduce the development of ADA during multiple doses of *E*. *coli* asparaginase.

In patients receiving replacement therapy, a significant factor affecting their risk to ADA development is the levels of endogenous protein, with patients expressing no or very little protein being at a much higher risk, presumably owing to compromised central tolerance induction (40). Even a few amino acid sequence changes between the endogenous protein and the administered biotherapeutic may lead to an increased risk in immunogenicity. Substitution of just three amino acids in the recombinant activated factor VII (rFVIIa) (1, 41) was shown to significantly increase immunogenicity of the therapeutic protein. In addition, dosing (42), protein folding/aggregation, route of administration, storage conditions, and excipients may also affect the development of ADA (43, 44). It has been proposed that even codon usage of the recombinant protein may affect protein conformation and modulate immunogenicity (45). The inhibitory activity of ADA can be mediated by several mechanisms. Development of anti-idiotypic antibodies against the therapeutic could lead to *in vivo* formation of immune complexes (ICs), which can diminish therapeutic efficacy by reducing the half-life of the therapeutic or engaging the complement cascade (46, 47). Larger ICs are removed from circulation faster than smaller ICs owing to engagement of FcR on macrophages, reducing drug levels and requiring more frequent administration (47, 48). Complement cascade activation (as seen with administration of therapeutic IFN-β for multiple sclerosis) enhances inflammatory responses (46, 47). Alternatively, generation of neutralizing antibodies (i.e., adalimumab and infliximab, anti-TNFα, and monoclonal Abs) could directly block the action of the administered antibody or modulate its *in vivo* half-life (18, 25, 49, 50). In rare cases, ADA generation may lead to anaphylactic shock and death (51).

### Lymph Nodes: Primary Sites for the Development of Immune Responses Against Pathogens

#### Structure

**Figure 1:**
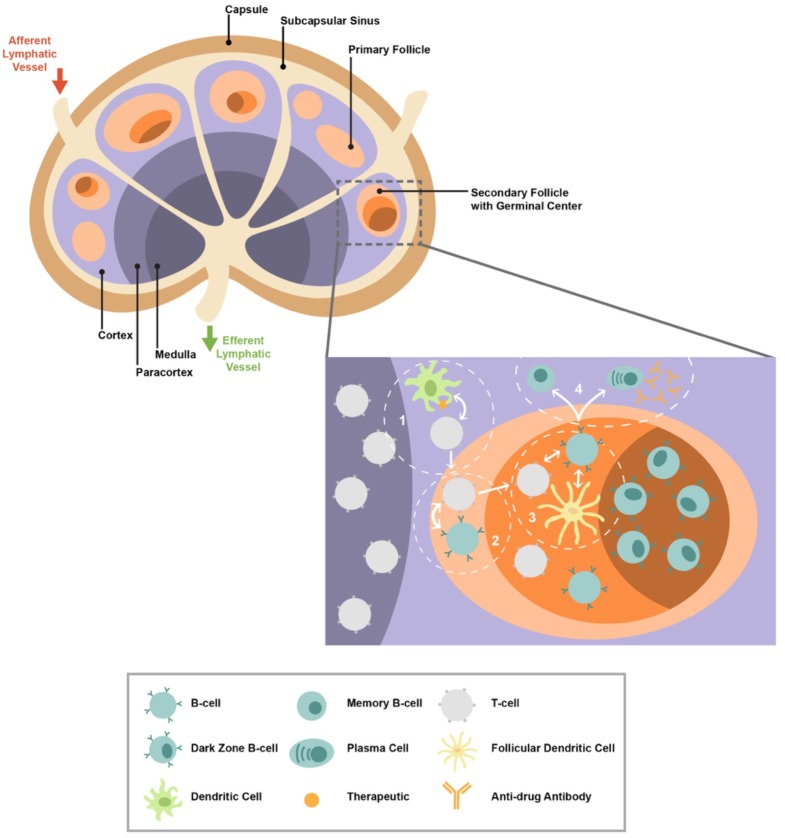
The lymph node structure/organization is shown. A zoomed T cell/follicular area with the major cell types involved in the development of antibody responses is shown. The presence of therapeutic within the lymph node can initiate a cascade of immune reactions ultimately leading to T cell-dependent germinal center (GC) activity and the generation of plasma cells and memory B cells that can produce antibodies. The cascade begins with (1) dendritic cells that present the therapeutic interaction with CD4 T cells resulting in their activation and differentiation; (2) activated CD4 T cells begin interacting with B cells, ultimately leading to further differentiation of both cell types and therefore trafficking into follicles/GCs; (3) within the GC, follicular CD4 T cells interact with GC B cells and follicular dendritic cell (FDC); (4) helping B cells promotes their maturation to memory and plasma cells.

Lymph node positioning along lymphatic vessels enables the efficient draining and detection of pathogens and immunogens (Figure 1). The number of human LNs varies depending on age and disease status (52–56). The LN architecture is characterized by well-organized, distinct anatomical areas: cortex, paracortex, follicles, germinal centers (GCs), high endothelial venules (HEVs), medulla, and fibroblastic reticular cells (FRCs) (57, 58) (Figure 1). The formation of distinct LN areas contributes to the compartmentalization of cellular and molecular mechanisms involved in the generation of antigen-specific humoral responses. This compartmentalization further contributes to the control of relevant immune interactions and reduction of unwanted B cell responses. The cortex consists of many lymphocytes, mainly naive B cells (sIgD+IgM+) packed into primary follicles (absence of GC) or secondary follicles that are characterized by the formation of GC (58, 59). GCs are the areas where B cells proliferate in response to T cell-dependent antigen and create memory cells and plasma cells (57). Two major GC areas have been characterized, dark zone (DZ) and light zone (LZ), with different cellularities and roles for the development of B cell responses (60, 61). The deeper cortex, also known as the paracortex, contains HEVs, which are specialized blood vessels that allow circulating lymphocytes, such as T cells, and innate immunity cells to directly enter the LN (58). The local interaction between T and dendritic cell (DC) subsets initiates a cascade of immune reactions critical to the formation of mature GCs (57). The medulla, located on the efferent side where the lymph drains out of the LN, contains blood vessels and medullary cords enriched in B cells, macrophages, and plasma cells (Figure 1). Finally, the backbone of the LN architecture is the FRCs. The FRCs form a network that allow DCs and T cells to travel throughout the LN (62).

#### Major Cell Populations

T cell zone (paracortex) is populated with innate immunity cells (DC, monocytes, macrophages, and granulocytes), adaptive immunity cells (CD4 and CD8), and stromal cells (FRCs). Subcapsular sinus macrophages is the first lymph node population encountering pathogens from the lymph (63) that controls the pathogen dissemination and inflammation and affects B cell responses to subsequent infections (64). These cells can trigger responses to lipid antigens, a mechanism mediated by activation of LN invariant natural killer cells (iNKTs) (65). Recirculating monocytes can traffic to LNs and either keep their classical status (66) or further differentiate to macrophages or DCs and initiate adaptive responses (67, 68). T cell zone macrophages can also function as scavengers for apoptotic cells (69). DC and monocyte in LNs are main producers of IL-6, an important cytokine for the differentiation development of Tfh cells (70, 71). FRCs provide a vital network for (i) recruitment of naive T cells and DCs through CCL21 and CCL19, the CCR7 ligands (72), a major chemokine receptor mediating tissue trafficking of several cell types (73, 74); (ii) T cell survival through IL-7, a survival factor particularly for naive T cells (75, 76); and (iii) trafficking of CD4 T cells toward the GC (62). When an antigen is present, major rearrangements take place within this area. Studies using mouse models have shown that the presence of antigen triggers the activation and repositioning of DC cells (77, 78), which have an important effect on CD4 T cell activation as well as the initiation of CD4 T and B cell interaction. B cells will follow a CCR7 gradient toward the follicle/T-zone (T–B) boundary where they could bind to multiple helper CD4 T cells at once, whereas T cells would only bind one at a time (79). The interaction of CD4 T and B cells in the T cell zone will activate a cascade of immune dynamics associated with major changes in differentiation status (phenotypes, transcriptome profile, and trafficking) (80) of both CD4 and B cells, ultimately enabling their trafficking into the GC area. In fact, the interaction between CD4, B, and antigen-presenting cells (APCs) in the T cell zone and T–B borders is required for optimal differentiation of CD4 T cells to Tfh cells (81, 82). These early interactions are also critical to the further development of B cell responses (83, 84).

## Germinal Center: The Laboratory for the Development of B Cell Responses

The role of the Tfh cells is to help create high-affinity memory B and plasma cells (85); thus, this subset of CD4 T cells is crucial in the immune response. GCs, the structures found in mature, secondary follicles (59), are populated with activated B cells, follicular DCs (FDCs), Tfh cells, and macrophages (tingible body macrophages) (59). Upon antigen stimulation, naive B cells traffic to the T–B border following a CCR7 gradient (79). Further interaction with CD4 T cells and receipt of co-stimulatory signals (86) trigger a rigorous proliferation of B cells and the formation of a tight cluster within the follicle, which becomes the GC. Within the GC, the B cells undergo somatic hypermutation, affinity maturation, class switch recombination, and plasma/memory B cell production (87–89). Formation of GC is mediated by help from FDCs and the function of G-protein-coupled receptors (GPCR) like S1PR2 and P2RY8 (59, 90, 91). In primary follicles, FDCs help B cells form a follicle (92), whereas in secondary follicles, FDCs support GC B cell survival (93–97). B cell survival was impaired when FDCs were exposed to HIV-1 (98), smaller GCs, formed and lower antibody titers were obtained when FDC activation was blocked through TLR4 (99). FDCs modulate antigen availability by cycling the antigens between the FDC surface and other endosomal compartments (100) or accumulating ICs bound to Fc receptors on their cell surface (100), a process critical to the affinity maturation of B cells (101, 102). Tfh cells are a subset of CD4 T cells that are specialized to help B cells. They are located inside B cell follicles of secondary lymphoid organs and are responsible for activation, isotype switching, affinity maturation, and differentiation of B cells (103–105). Tfh cells express a unique phenotype and transcriptome signature (106–109). A mutual regulation through modulation of Bcl-6 between Tfh and GC B cells has been proposed (110–112). Tfh cells produce cytokines like IL-21 and IL-4, which are important for the GC B cell dynamics (105, 113–115) and maintenance of Tfh cells. Two distinct GC areas have been identified; the DZ, where B cell proliferation and somatic hypermutation occurs, and the LZ, where B cells interact with Tfh cells and FDCs (60, 116). Expression of the chemokine receptor CXCR4, which is highly expressed on Tfh and GC B cells (117, 118), and local production of its ligand CXCL12 (SDF-1) in DZ by CXCL12-expressing reticular cells (CRC) (119, 120) play a critical role for the organization of DZ and LZ (116, 121, 122). LZ is less compact and more diverse than is DZ. B cells continuously move between the DZ and LZ, helping in the further differentiation and affinity maturation of GC B cells (123–126). Within the LZ, B cells continuously interact with Tfh, FDC, and antigen, interactions that dictate their survival and clonal selection (84, 127–129).

### Germinal Center Reactivity Against Therapeutics

Several studies have investigated GC dynamics after drug administration. Understanding how the LN and GC react to therapeutics (antibodies, recombinant proteins, cytokines, vaccines, enzymes, etc.) is important to reduce/eliminate ADA development. ADA can be generated by T cell-dependent (Td) and T cell-independent (Ti) pathways (130–132). The Td pathway involves an antigen-activated T cell that then stimulates B cell activation and differentiation to plasma cells. Neutralizing IgG4 ADA against FVIII (6, 133) was triggered by a Th2 CD4 T cell response (134), whereas initiation of Th1 responses was found to induce IgG1 and IgG2 ADA against FVIII when patients were on immunosuppressive therapy (134), which may sometimes be non-neutralizing (133, 134). Administration of IFN-β was found to induce either non-neutralizing and transient neutralizing ADA, mainly of low titers and affinity IgG1 and IgG3 subclasses, or persistent neutralizing ADA, which had mostly IgG2 and IgG4 antibodies (135). ADA binding affinity was positively correlated with IgG4 production and neutralizing ADA titers but negatively correlated with IgG3 production. Similarly, generation of high-affinity antibodies to biopharmaceuticals is CD4 T cell dependent (136, 137). In fact several studies have shown the development of neutralizing antibodies ADA (138–142) mainly of IgG4 subtype (143). Similar polyclonal IgG1 responses consisting of neutralizing and non-neutralizing specificities have also been detected in response to natalizumab (NZM) administration in multiple sclerosis patients. Neutralizing antibodies in these patients carry a higher load of somatic mutations in the complementary determining regions (CDRs) and have a higher affinity than have non-neutralizing, binding antibody specificities, which is consistent with LN-specific antigen-driven selection (144).

These considerations indicate that alternative cytokine milieu and initial programing of CD4 T cells in the LN can affect the outcome of the GC B cell responses to a given therapeutic in a way that parallels the LN-associated changes seen in vaccine-specific or pathogen-associated antibody production. The Ti pathway is triggered when the B cell is activated directly by the antigen. In general, polyvalent antigens, such as an aggregated biologic (145, 146), are more likely to induce Ti-B cell responses (147, 148). Ti responses lead to IgM or low affinity IgG ADA owing to lack of T cell help (130). Because most ADAs are IgG, the possible role of complement activation by ADA needs further investigation (130). Neutralizing antibodies, particularly the broad neutralizing antibodies (bNABs), are characterized by high levels of somatic mutations (149) and are indicative of GC maturation. The mechanisms leading to such maturation process in the GC are not well-understood. Presumably, antigen concentration within LN/GC and the co-evolution of Tfh and GC B cells (selection of TCR and BCR clones) are major biological factors affecting this process. Studies using mouse models have shown that the quality of Tfh help to GC B cell is an important biological factor for the development of high-affinity antibodies (127, 129, 150). Specifically, Tfh helps regulate the metabolic programming of LZ B cells that support their proliferation in DZ (129). Furthermore, this helps prolong the duration of B cell cycle in the DZ, a process associated with the generation of high-affinity GC B cells (151). Shuttling/binding of antigen to FDC (152) as well as the amount of antigen presented to Tfh by GC B cells (150) can have a significant impact on the development of high-affinity B cell responses. Dynamics and factors in the follicular, non-GC area can also affect the maturation of B cell responses. High-affinity B cell clones can be selected during early interaction between CD4 T and B cells at the T–B cell border (84), whereas class switch recombination can be started outside GC (153). Furthermore, recently identified populations like the CD25+FoxP3+ T follicular IL-10-producing cells (154) and Tbet+ B cells, mainly localized around the GC (155), could be important regulators for the development of neutralizing antibodies. Host genetics are also relevant. The presentation of MHC class II-restricted drug-specific peptides on CD4+ T cells can further contribute to the emergence and maintenance of polyclonal drug-specific B cell responses in lymphoid localities (144). Conversely, elimination of specific drug-associated T cell epitopes in mice treated with recombinant immunotoxins curtails the development of high-affinity antidrug IgG responses in primary as well as anamnestic responses (156). Taken together, these findings suggest that at least for some types of biologic pharmaceuticals, T-dependent pathways in LNs are central in the induction of neutralizing ADAs. Therefore, understanding in more detail the nature, trafficking/distribution of each biopharmaceutical into LN and its availability/sustainability on FDCs is warranted, as these are factors could direct the cellular and molecular mechanisms immobilized in the LN, which lead to the development of specific types of antibody responses, especially in the absence of adjuvants, which trigger innate immunity.

## Conclusions

The cellular and molecular mechanisms governing the development of ADA responses in humans are not well-understood. This is a highly coordinated process taking place in secondary lymphoid organs where the nature of the “antigen,” tissue structure, and spatial positioning of relevant cell populations, particularly in the follicular/GC area, play a critical role for the host–therapeutic interplay leading to the differentiation of adaptive immune cells to enable the generation of antibody-secreting B cells. Today, the link between ADA and changes in LN function is still not well-studied. Major aspects related to ADA development that need further investigation include the following:

1. The trafficking and sustainability of a particular therapeutic in the LN areas, particularly the GC. Formulation for the *in vivo* delivery of an immunogen could significantly change its dynamics in the GCs with major impact on the B cell response development (157, 158).
2. The activation of specific innate immunity cells and the concomitant changes in the local cytokine/chemokine milieu are factors regulating the degree of CD4 T cell help for the B cell responses (78).
3. The possible association between ADA titer and affinity maturation and particular GC dynamics (i.e., magnitude of Tfh cell responses and expansion of particular Tfh subsets).
4. The possible role of preexisting immune activation and inflammation within the lymphoid organs.

Of particular interest is the investigation of ADA development in aging where the GC dynamics are different compared with those in young individuals (159–161) as well as in chronic inflammatory diseases like HIV and autoimmunity. For example, altered antibody responses are expected in HIV-infected individuals where chronic infection is associated with LN inflammation, architecture damage (fibrosis), and dramatic changes to GC dynamics (100, 155, 162, 163). Such LN changes could have a major impact in the ADA development to a specific therapeutic. Despite the limited predictive value for the drug immunogenicity in humans based on non-human primate (NHP) studies (164), NHP represents the only model for testing antibody development under such conditions. However, we need to keep in mind that compared with that in humans, ADA in NHP is mainly directed against the Fc region of the monoclonal antibody, causing loss of efficacy and adverse effects. Supplemental to human studies, investigation of therapeutic immunogenicity, when it occurs, in NHPs could lead to identification of particular cell types, molecules, and molecular pathways driving the responses to a particular therapeutic. The wide range of titers, subtypes, and function of ADA induced by different therapeutics argues for the need for identification of “LN molecular/cellular signatures” specific to certain therapeutic, which could lead to targets for “individualized” *in vivo* manipulation of ADA development.

## Author Contributions

All authors listed have made a substantial, direct and intellectual contribution to the work, and approved it for publication.

## Conflict of Interest

The authors declare that the research was conducted in the absence of any commercial or financial relationships that could be construed as a potential conflict of interest. The handling editor declared a shared affiliation, though no other collaboration, with one of the authors AA at time of review.
